# Cerebral Venous Sinus Thrombosis Associated With Contraceptive Use: A Case Report

**DOI:** 10.7759/cureus.86576

**Published:** 2025-06-23

**Authors:** Syndy Guarin-Rivera, María Gil-Lancheros, Cristian Castillo-Ramírez, Jesus Utria-Munive

**Affiliations:** 1 Internal Medicine Service, Méderi Barrios Unidos University Hospital, Bogota, COL; 2 Epidemiology and Biostatistics, University Foundation of Health Sciences (FUCS), Bogota, COL

**Keywords:** cerebral venous thrombosis, headache, oral contraceptives, thrombosis, women

## Abstract

Cerebral venous thrombosis (CVT) is a condition that may affect women of childbearing age and is, in some cases, associated with risk factors such as oral contraceptive use. This case describes the presentation of a case of cerebral venous sinus thrombosis in a woman of childbearing age who uses oral contraceptives. A 28-year-old woman presented to the internal medicine service with a clinical picture of intense right temporo-occipital headache of eight days of evolution associated with flashes of light and vertigo without improvement with analgesics. She has a history of use of cyproterone acetate and ethinylestradiol for acne management initiated one month prior, with cerebral MR venography revealing venous thrombosis of the right transverse, petrosal, and sigmoid sinuses, requiring anticoagulation with low-molecular-weight heparin (LMWH). Autoimmune diseases and thrombophilia were ruled out. Therefore, CVT can be considered in the differential diagnosis of all headaches of recent onset and refractory to treatment, especially in women of childbearing age, with cerebral resonance angiography being essential for the diagnosis. Anticoagulation is the cornerstone of treatment.

## Introduction

Cerebral venous thrombosis (CVT) is a rare and potentially fatal form of stroke that corresponds to 0.5-1% of strokes globally [1-4]. Its incidence varies between two and five cases per 1,000,000/adult population [5], although an increase of up to 10 times this figure has been reported due to advances in neuroimaging techniques that allow the early detection of the disease [4]. CVT occurs most frequently in young adults (20-35 years) and in women, especially in women of reproductive age, which has been related to pregnancy, puerperium, and oral contraceptive pill (OCP) use [6].

The etiology of CVT is multifactorial and can be septic or aseptic, the latter being the most frequent at present. Among the multiple causes described, there are systemic inflammatory diseases, pregnancy and puerperium, neoplasms, use of OCPs, coagulation disorders, hematological alterations, trauma, and infections, among others. However, about 15-20% of cases do not have a clear etiology [3,4].

The symptoms are diverse, depending first of all on the site and extent of the thrombosis, as well as the underlying pathophysiological mechanism: increased intracranial pressure due to alterations in venous drainage or cerebral ischemic injury due to decreased cerebral perfusion [4,7]. Thus, the clinical presentation can range from isolated headache to frank neurological alterations, such as motor or focal sensory deficit, papilledema, changes in mental status, seizures, and even sudden death [1]. This broad spectrum of symptoms can make diagnosis and initiation of treatment difficult and delayed [5]. This report aims to describe the clinical case of a young woman who developed cerebral venous sinus thrombosis associated with the use of combined oral contraceptives, highlighting the importance of early recognition of risk factors and timely clinical management to avoid severe complications in patients with this pathology.

## Case presentation

A 28-year-old woman with a normal body mass index, with no reported stressors or head trauma, presented to the emergency department with an eight-day history of right temporo-occipital headache radiating to the ipsilateral hemiface. The pain was moderate to severe in intensity and associated with nausea, intermittent episodes of vertigo, and flashes of light, with no vomiting, no flu-like symptoms, or fever. During the initial assessment by General Medicine, physical examination revealed bulging of the tympanic membrane, leading to a presumptive diagnosis of otitis media. She was treated with acetaminophen-codeine, naproxen, and amoxicillin-clavulanate. However, upon follow-up with Otolaryngology, the diagnosis of otitis media was ruled out, and the prescribed treatment was discontinued. She had an increase in headaches and insomnia, so she went to the emergency room again.

**Figure 1:**
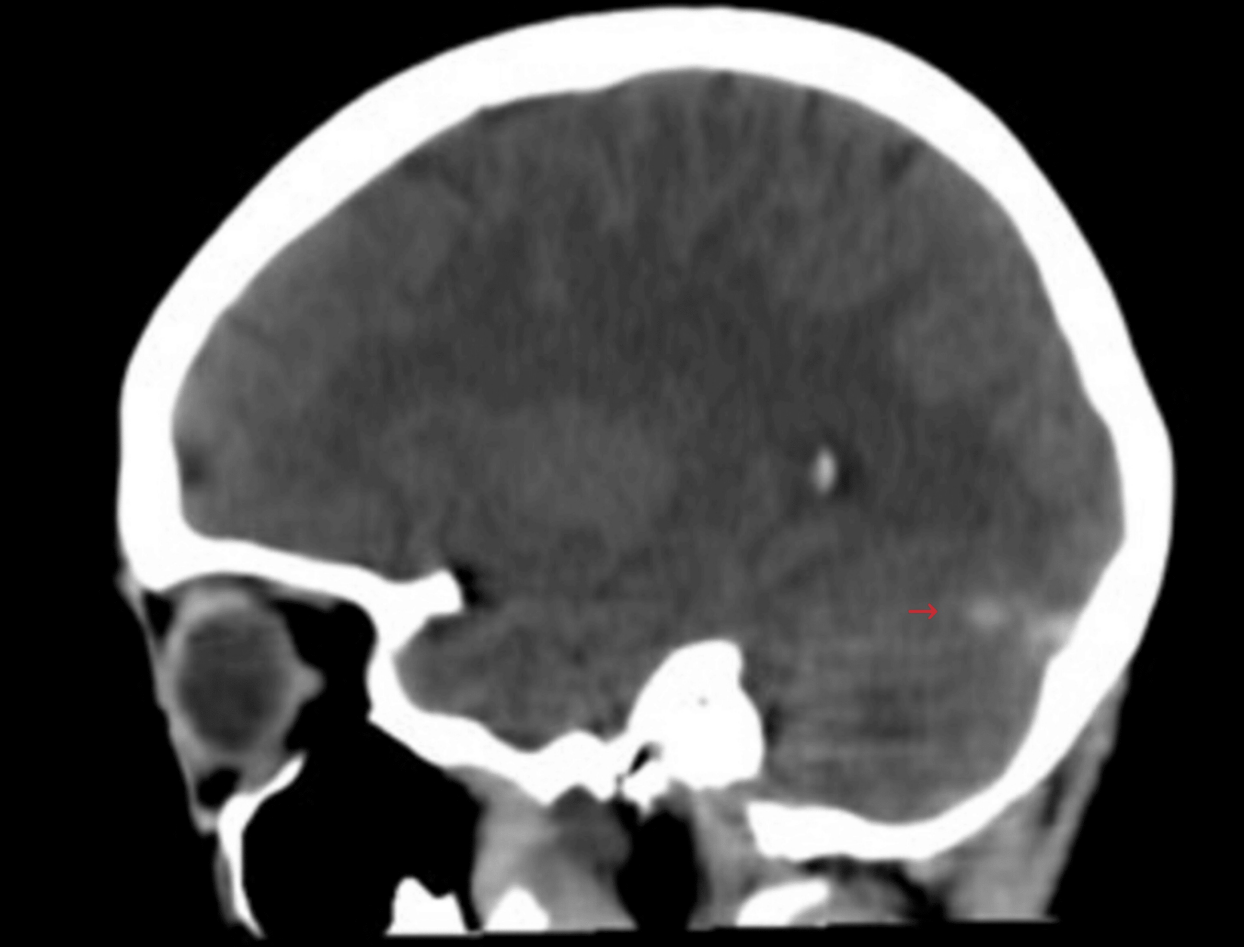
Sagittal CT slicing of the single skull Hyperdensity of the right transverse sinus

**Figure 2:**
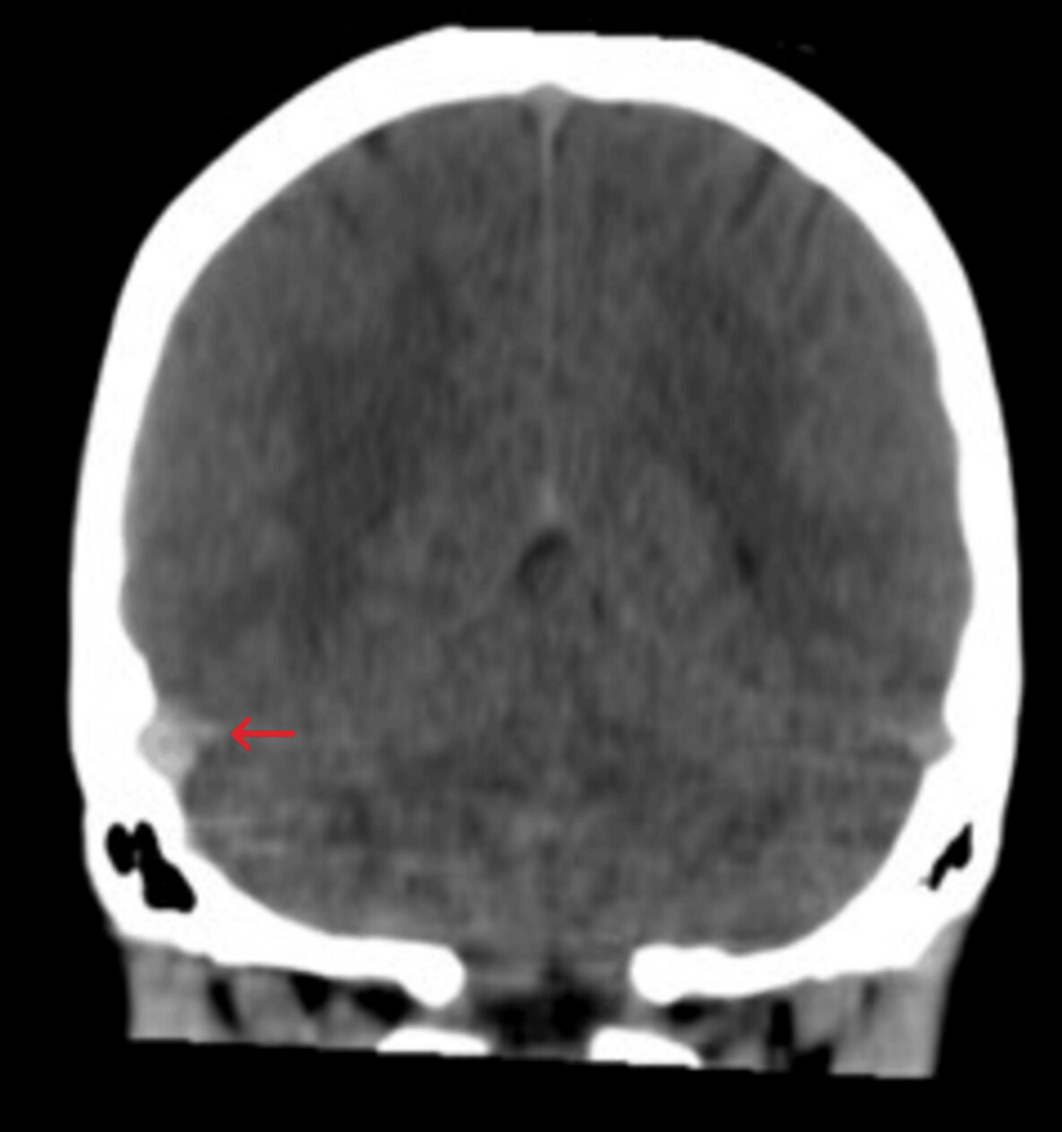
Coronal CT scan of the single skull Hyperdensity at the level of the right transverse sinus

**Figure 3:**
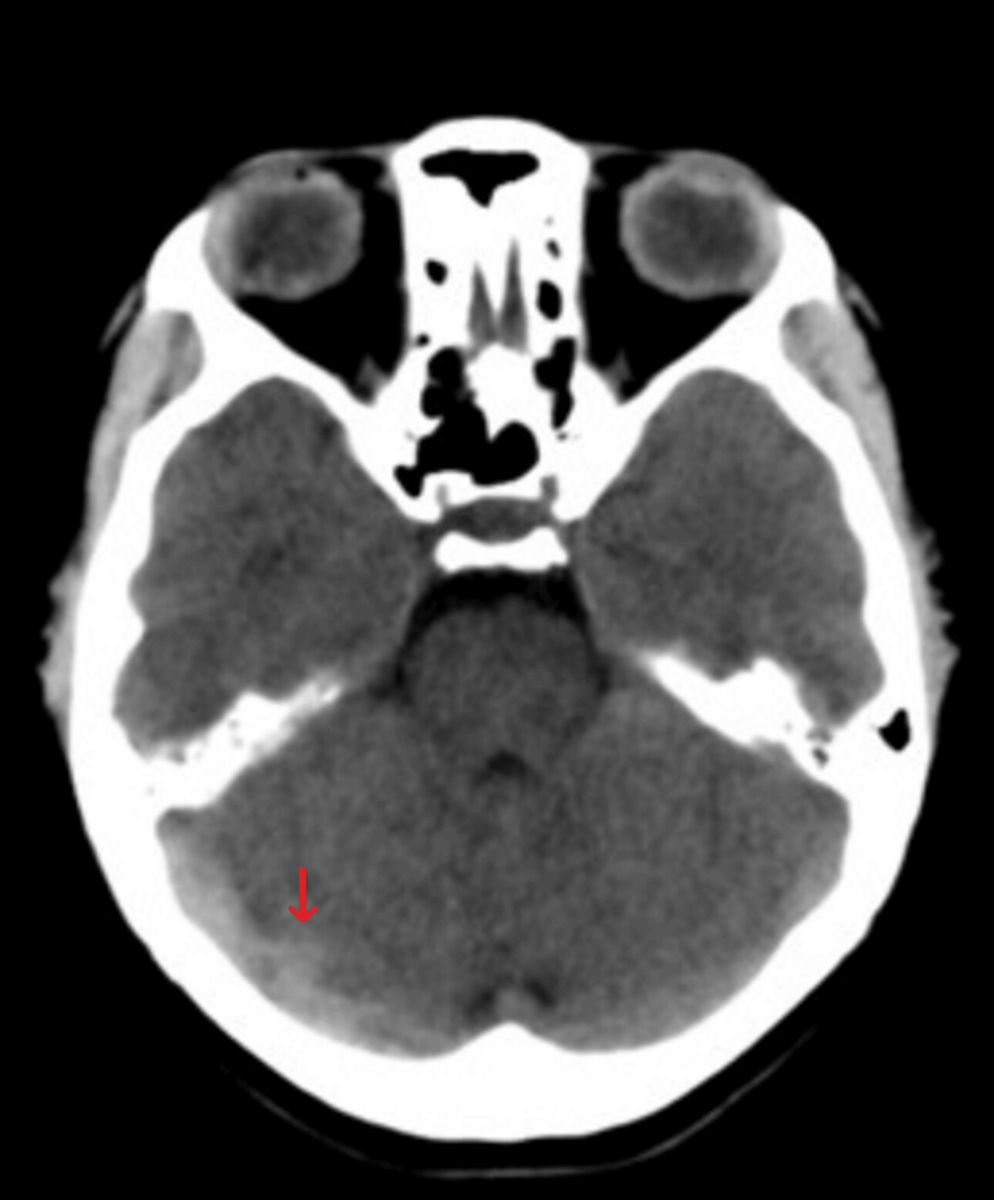
Axial CT scan of the single skull Hyperdensity in the right transverse sinus pathway

**Figure 4:**
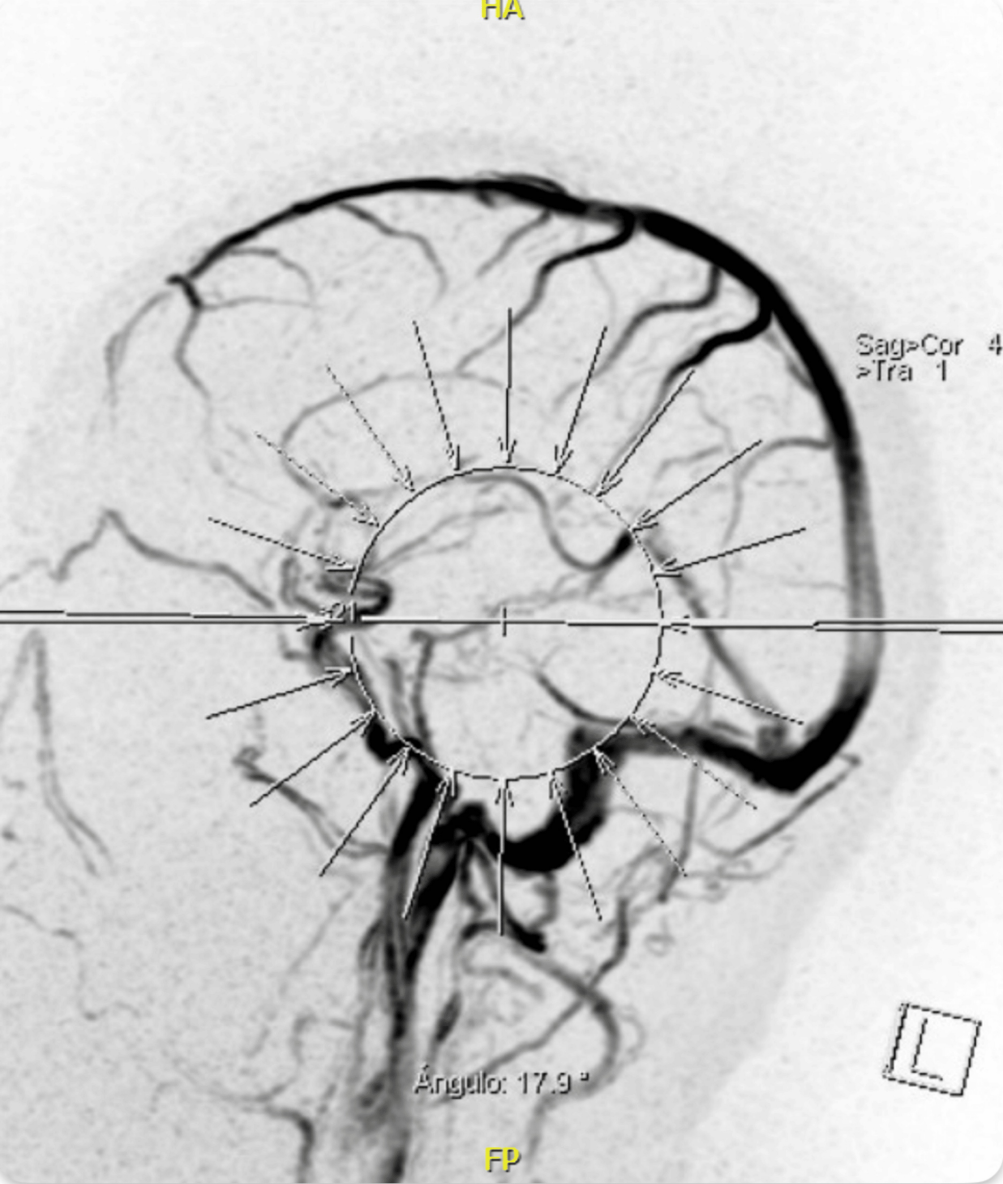
Sagittal section of the brain Absence of flow from the right transverse and sigmoid sinuses

**Figure 5:**
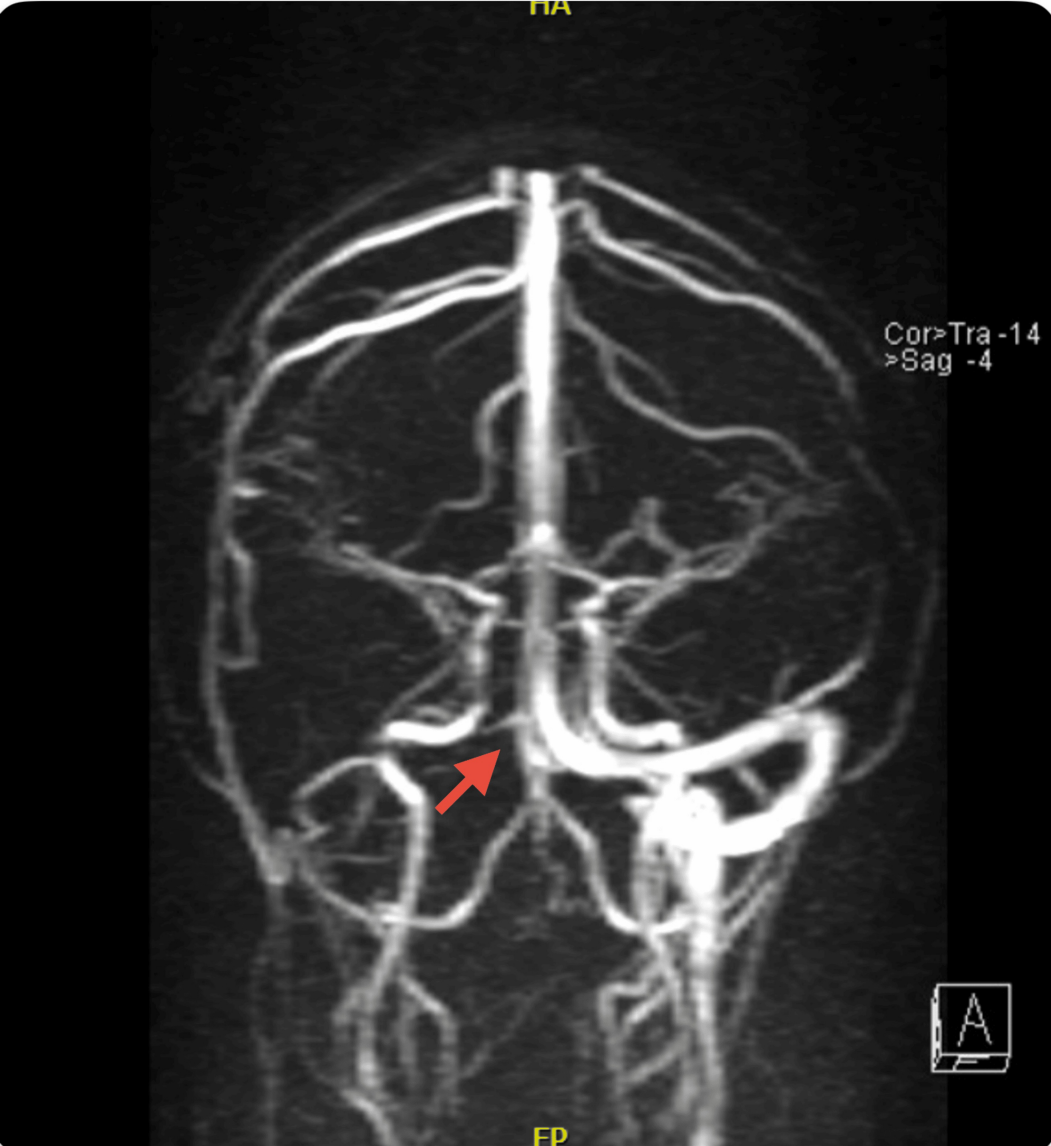
Coronal section of the brain Absence of flow from the right transverse sinus

On admission, she had blood pressure within normal limits and was not tachycardic or febrile; she showed no signs of focalization, involvement of low, meningeal or cerebellar cranial nerves, or alterations in postural tone or gait; Romberg's sign was negative. She was prescribed a normal diet without caffeine; administration of isotonic saline solution at 1 cc/kg/hour, prophylactic anticoagulation with low-molecular-weight heparin subcutaneously every 24 hours, paracetamol 1 gram orally every eight hours, omeprazole 20 mg orally daily, and morphine 2 mg intravenously every six hours was indicated. A simple cranial tomography was requested that showed "unusual hyperdensity in the right transverse sinus" (Figures 1, 2, 3), so a cerebral resonance angiography was performed in the venous phase, revealing "acute thrombosis of the right transverse, petrosal, and sigmoid venous sinuses" (Figures 4, 5). Therefore, anticoagulation was adjusted to low-molecular-weight heparin at a dose of 1 mg/kg.

Internal Medicine evaluated the patient, finding pain of 5/10 intensity, no fever or irritative urinary symptoms, no malar erythema, no joint pain, and no bleeding or skin lesions. The neurological examination showed no changes compared to the initial assessment upon admission. The medical history revealed a history of gastritis, irritable bowel syndrome, migraine, and acne, the latter treated with isotretinoin (20 mg/day) four months ago and cyproterone with ethinyl estradiol (2/0.035 mg daily) during the last month. The last menstruation was on 08/11/23, with regular cycles and without previous pregnancies or abortions. Family history included high blood pressure, type 2 diabetes mellitus, and dyslipidemia, with no history of thrombophilia or autoimmune diseases.

It was considered that thrombosis could be secondary to the use of oral contraceptives due to the temporality between exposure and the occurrence of the thrombotic event. Additional studies were requested that ruled out infections, autoimmune diseases, and thrombophilia, although there was a slight prolongation of the lupus anticoagulant, considered not significant due to a negative Russell's viper time and possibly associated with the use of anticoagulants (Tables 1-6).

**Table 1:** Blood count and their reference values

	Laboratory findings			Reference value
	Day 1 First assessment	Day 1 Second assessment	Day 3	
Leukocytes (mm^3^)	12089	12780	8950	4000-10000
Neutrophils (%)	74.1	85.6	64.1	40-60
Lymphocytes (%)	20.1	11.3	29	20-40
Hemoglobin (gr/dl)	15.6	16.4	15	14-16
Hematocrit (%)	47.5	49.3	45.1	38-50
Platelets (mm^3^)	274000	292000	251000	150.000-450.000

**Table 2:** Electrolytes and their reference values

Laboratory	Day 1	Day 3	Reference value
Sodium (mmol/L)	135.9	137.60	135-145
Potassium (mmol/L)	4.20	4.15	3.5-5.1
Calcium (mg/dL)	8.4	8.32	8.5-10.1

**Table 3:** Renal function and their reference values BUN: blood urea nitrogen

Laboratory	Day 1	Day 3	Reference value
BUN (mg/dL)	12.4	13	7-21
Creatinine (mg/dL)	0.79	0.89	0.57-0.8

**Table 4:** Serological tests †RPR: rapid plasma reagin, *HIV: human immunodeficiency virus

Laboratory	Day 3
RPR serology †	Non-reactive
HIV 1-2 antibodies	Negative
Hepatitis B (surface antigen)	Non-reactive

**Table 5:** Coagulation times and their reference values ‡TP: prothrombin time, §TPT: partial thromboplastin time; ¶INR: international normalized ratio

Laboratory	Day 3	Day 6	Day 8	Day 11	Reference value
PT (sec) ‡	10.7	10.6	10.7	18.5	9.9-11.8
TPT (sec) §	23	26.2	-	-	25-31.3
INR ¶	1	0.99	1	1.79	

**Table 6:** Autoimmune profile and their reference values ** international unit

Laboratory	Day 3	Reference value
Beta 2 glycoprotein 1 IgG (International Unit/ml)	1.9	Positive: greater than 8
Beta 2 glycoprotein 1 IgM (IU/mL)	2.7	Positive: greater than 8
Cardiolipins IgG Antibodies GPL (IU/mL)	1.4	Positive: greater than or equal to 10
Cardiolipins IgM MPL Antibodies (IU** /ml)	1.8	Positive: greater than or equal to 7
Serum C3 supplement (mg/dL)	139.39	82-160
C4 serum supplement (mg/dL)	31.35	12-36
Lupus anticoagulant PTT-LA (sec – presumptive test)	39	32.7
Rheumatoid factor IU/ml	<3.5	<14
Antinuclear antibodies (ANA)	Negative	
Time on Russell's Viper Venom Radio LA (LA1/LA2)	1.1	<1.20

In the neurological evaluation, it was indicated to continue with analgesic management (paracetamol, morphine, and topiramate) and anticoagulation, performing a lumbar puncture due to the regular persistence of the headache. This procedure showed evidence of intracranial hypertension, but it was carried out without complications, and the cerebrospinal fluid findings were normal (Table 7).

**Table 7:** Cerebrospinal fluid *** PMN: polymorphonuclear; § ADA: adenosine deaminase; **VDRL: Venereal Disease Research Laboratory

Type of analysis	Day 6
	Laboratory findings
Chemical analysis	Color: colorless
	Appearance: limpid
	PH: 7.0
	Protein: 25.1 mg/dL
	Albumin: 0.01 gr/dL
	Glucose: 56.8 mg/dL
	Lactic dehydrogenase: 16.13 U/L
Microbiological analysis	Total white blood cell count: 0.0 mm3
	Mononuclear: 0.0%
	***PMN: 0.0%
	Erythrocytes: 0.0 mm3
	Fresh erythrocytes: 0.0%
	Crenate erythrocytes: 0.0%
	Growing common germs: Negative
	Indian ink coloring: negative
	Cryptococcus: negative.
	Gram coloration: no microbiota observed
	Serology (**VDRL): non-reactive.
	§ADA: 0.1 U/L
	Acid-fast staining: no acid-fast bacilli observed in 100 microscopic fields

Improvement in headache was documented during hospitalization, and after seven days of treatment, the patient was discharged with anticoagulant bridging treatment with enoxaparin (60 mg/12 hours) and warfarin (5 mg/day), with outpatient follow-up. Upon reaching the target INR (2.0-3.0) on the ninth day of discharge, enoxaparin was discontinued, and follow-up was continued in the anticoagulation clinic. The patient returned approximately one month after hospital discharge due to the recurrence of a headache in the right hemicranium. A new plain cranial tomography was performed, which showed no lesions suggestive of thrombosis or bleeding. The patient, with a neurological examination without alterations and normal campimetry, presented an INR within the objectives, so she was discharged with analgesia adjustment and outpatient evaluation by neuropsychology and occupational medicine was requested to investigate a possible relationship between the reappearance of the headache and the return to work.

**Figure 6:**
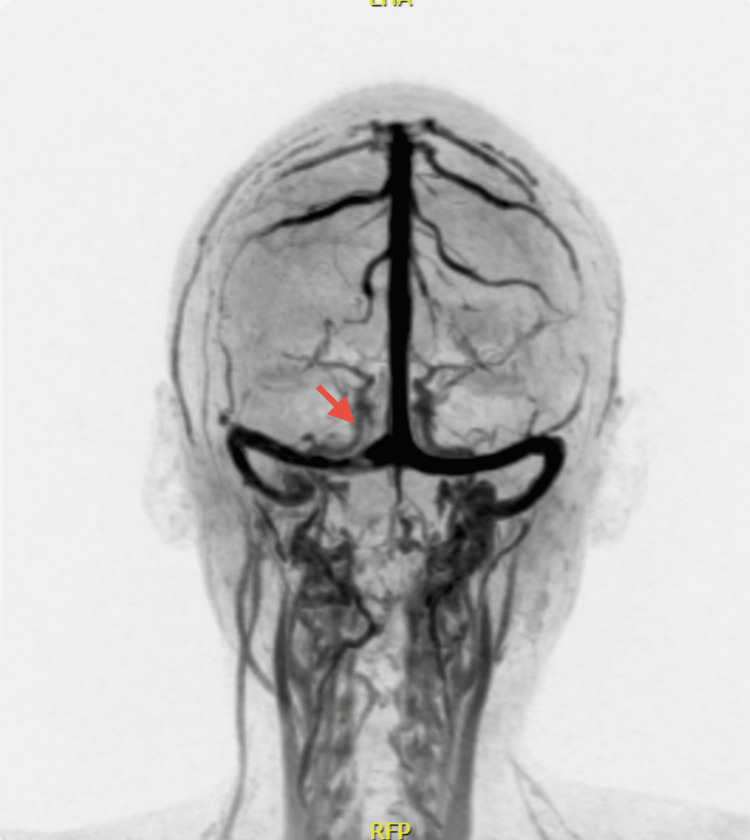
Coronal section of the brain Recanalization of the affected venous sinuses

**Figure 7:**
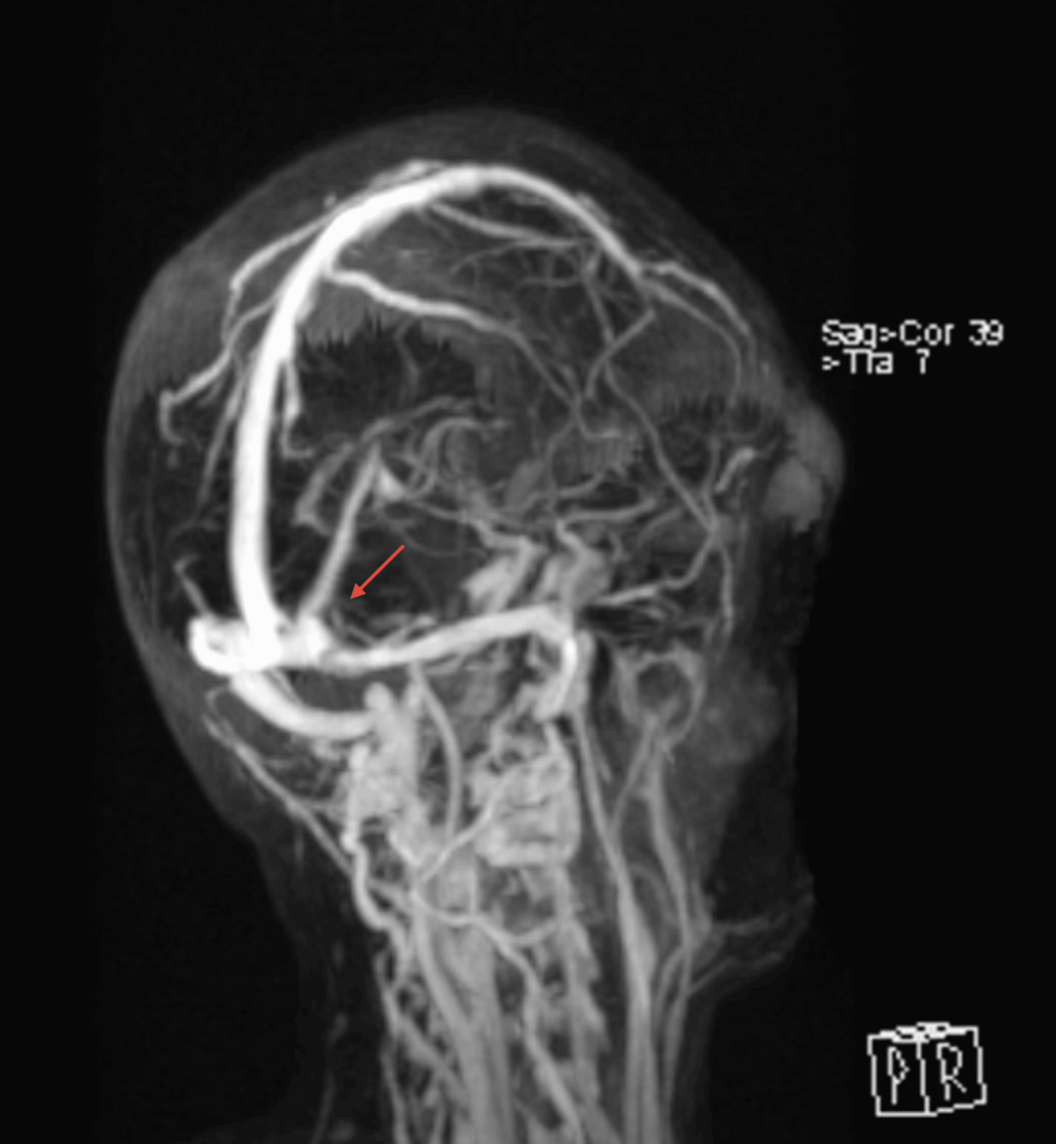
Sagital section of the brain Recanalization of the affected venous sinuses

During outpatient follow-up one month later, the patient reported an improvement in headache with the use of analgesics and working from home. Hematology replaced warfarin with rivaroxaban, and neurology recommended continuing with the anticoagulant management indicated by hematology and performing imaging controls. Three months later, ophthalmology ruled out any visual compromise. Based on the results of the paraclinical examinations and the measures taken during outpatient follow-ups (Figures 6, 7), the diagnosis of cerebral vascular thrombosis secondary to the use of oral contraceptives was established. 

## Discussion

We present the case of a woman of childbearing age with a normal weight who developed CVT after taking oral contraceptives for acne treatment. This case exemplifies the challenges involved in making an initial and timely diagnosis of CVT. Despite improvements in early detection due to advances in imaging, a high index of clinical suspicion remains essential because the symptoms are variable and nonspecific. CVT is a multifactorial disease, with at least one risk factor present in up to 85% of adults. Prothrombotic conditions were found to be more closely related [4]. CVT most commonly occurs in the transverse sinus (86%), followed by the superior sagittal sinus (62%), rectus sinus (18%), and cortical veins (17%) [8]. It is less frequently observed in the petrosal and sigmoid sinuses [9,10]. Symptoms of CVT can range from mild to severe, so for some patients, headache is the only relevant symptom [8]. However, the patient had acne, which is a chronic disease with variable clinical presentation that in up to 30% of cases will require pharmacological therapy, mainly with oral or systemic retinoids, oral antibiotics, isotretinoin, or hormonal contraceptive methods as joint management [11]. The adverse effects associated with the use of oral contraceptives are minimal compared to the expected benefits, with headache, mood variations, and intermenstrual bleeding standing out within this group [12]. Headaches can occur with the use of oral contraceptive pills (OCPs) without the development of thrombosis, so it is essential to have adequate questioning and physical examination to determine the possibility of this complication, since the delay in its diagnosis can lead to greater complications.

Thrombosis is among the most severe side effects associated with the use of oral OCPs, primarily due to the estrogen component in these medications. Progestogens also elevate the risk of thrombosis, although not as significantly as estrogens, particularly with third-generation progestogens during the initial three months of use [11]. It is important to note that the absolute risk of thromboembolism from oral contraceptive use in otherwise healthy individuals without a prior cardiovascular history is low and remains lower compared to the risk posed by the patients' healthy lifestyle practices. [13]. OCPs may also alter the results of prothrombotic tests and increase the likelihood of false positives during thrombophilia screening [8]. In addition, oral contraceptives may increase the risk of cardiovascular disease, breast cancer, and melanoma [14,15]. The use of OCPs is one of the consistently identified risk factors for the development of CVT, with the risk being described as increasing approximately sixfold compared to women who do not use this medication [5,7]; Therefore, if any neurological disorder is suspected in any woman of reproductive age, her medical history should be examined, especially if she is exposed to OCPs [2]. In the case of this patient, she had a time of oral contraceptive consumption of one month prior to symptoms.

Magnetic resonance angiography is recognized as a useful tool for detecting CVT [2]. Furthermore, CVT can be life-threatening, potentially leading to seizures, infections, and brain herniation caused by increased intracranial pressure. In certain cases, neurosurgical management is required, such as in the presence of an intracerebral hematoma with mass effect, obstructive hydrocephalus due to involvement of the deep venous sinuses, progressive neurological deterioration despite adequate anticoagulation, or extensive thrombosis with massive cerebral edema. In certain cases, neurosurgical management is required, such as in the presence of an intracerebral hematoma with mass effect, obstructive hydrocephalus due to involvement of the deep venous sinuses, progressive neurological deterioration despite adequate anticoagulation, or extensive thrombosis with massive cerebral edema [8]. The primary approaches to treating CVT include addressing the underlying causes, administering anticoagulant medications, and managing symptoms [2].

Anticoagulant therapy is a cornerstone in managing CVT, aiming to prevent thrombus expansion and facilitate the reestablishment of normal blood flow in the affected area [5,7]. The duration of anticoagulation typically ranges from three to 12 months, contingent on the risk of recurrence of the thrombotic event [8]. In our case, the patient was initially treated with warfarin, which effectively controlled her INR and alleviated her headache symptoms. Subsequently, due to negative thrombophilia studies, the hematology service switched warfarin to rivaroxaban, as the former had initially been used due to a lack of direct oral anticoagulant (DOAC) availability for hospital discharge. In addition, warfarin therapy was not continued in order to avoid its associated restrictions, such as dietary limitations. The stratification of the risk of CVT recurrence aims to pinpoint factors that elevate the likelihood of a new thrombotic event, necessitating timely and appropriate studies [7,16]. Overall, CVT tends to have a favorable prognosis when diagnosed and treated promptly, but a high degree of clinical suspicion is essential to guide proper management.

## Conclusions

In conclusion, this case highlights the importance of considering cerebral venous sinus thrombosis as a possible complication in young women using OCPs, especially when they have new-onset headaches that do not respond to standard treatment. Early detection using neuroimaging techniques, such as magnetic resonance angiography, and the timely implementation of anticoagulant treatment are essential to improve the prognosis of these patients and prevent serious complications. In this case, anticoagulant treatment allowed a favorable evolution without recurrence of thrombosis. However, it is crucial to maintain close follow-up to adjust anticoagulant management and avoid recurrences, underlining the need to raise awareness of the risks associated with the use of OCPs in patients with predisposing factors.

## References

[1] Guenther G, Arauz A (2011). Cerebral venous thrombosis: a diagnostic and treatment update. Neurologia.

[2] Ferro JM, Aguiar de Sousa D (2019). Cerebral Venous Thrombosis: an Update. Curr Neurol Neurosci Rep.

[3] Stam J (2005). Thrombosis of the cerebral veins and sinuses. N Engl J Med.

[4] Idiculla PS, Gurala D, Palanisamy M, Vijayakumar R, Dhandapani S, Nagarajan E (2020). Cerebral Venous Thrombosis: A Comprehensive Review. Eur Neurol.

[5] Capecchi M, Abbattista M, Martinelli I (2018). Cerebral venous sinus thrombosis. J Thromb Haemost.

[6] Plewa MCT, Prasanna. Gupta, Mohit Mohit Cavernous Sinus Thrombosis. StatPearls2023. https://www.ncbi.nlm.nih.gov/books/NBK448177/.

[7] Saposnik G, Barinagarrementeria F, Brown RD Jr (2011). Diagnosis and management of cerebral venous thrombosis: a statement for healthcare professionals from the American Heart Association/American Stroke Association. Stroke.

[8] Ferro JM, Canhão Pa Cerebral venous thrombosis: Etiology, clinical features, and diagnosis. https://www.uptodate.com/2023..

[9] Duman T, Uluduz D, Midi I (2017). A Multicenter Study of 1144 Patients with Cerebral Venous Thrombosis: The VENOST Study. J Stroke Cerebrovasc Dis.

[10] Delgado-Reyes L, Pérez-Torres A, Gasca-González OO, Pérez-Cruz JC (2023). Seno cavernoso: anatomía, histología y terminología. Cir Cir.

[11] Requena C, Llombart B (2020). Oral Contraceptives in Dermatology. Actas Dermosifiliogr (Engl Ed).

[12] Sigindioy CY, Velásquez ATT, Díaz CCS (2021). Anticonceptivos hormonales y sus efectos adversos en mujeres de 18 a 25 años de la ciudad de Bogotá. Revista CIES Escolme. http://revista.escolme.edu.co/index.php/cies/article/view/353.

[13] de Bastos M, Stegeman BH, Rosendaal FR, Van Hylckama Vlieg A, Helmerhorst FM, Stijnen T, Dekkers OM (2014). Combined oral contraceptives: venous thrombosis. Cochrane Database Syst Rev.

[14] Bhupathiraju SN, Grodstein F, Stampfer MJ, Willett WC, Hu FB, Manson JE (2016). Exogenous Hormone Use: Oral Contraceptives, Postmenopausal Hormone Therapy, and Health Outcomes in the Nurses' Health Study. Am J Public Health.

[15] Roethlisberger M, Gut L, Zumofen DW (2018). Cerebral venous thrombosis requiring invasive treatment for elevated intracranial pressure in women with combined hormonal contraceptive intake: risk factors, anatomical distribution, and clinical presentation. Neurosurg Focus.

[16] Estellés Cortés A, Gilabert-Estellés J (2001). Anticonceptivos hormonales orales, coagulación y trombosis. Revista Clínica Española.

